# Comparing the Effect of Moderate-Intensity Versus High-Intensity Interval Training Exercise on Global Longitudinal Strain (GLS) in Cardiovascular Patients: Systematic Review and Meta-Analysis

**DOI:** 10.1155/crp/9901472

**Published:** 2025-02-10

**Authors:** Saeed Ghazavi, Reihaneh Zavar, Masoumeh Sadeghi, Afshin Amirpour, Atefeh Amerizadeh

**Affiliations:** Department of Cardiac Rehabilitation, Cardiac Rehabilitation Research Center, Cardiovascular Research Institute, Isfahan University of Medical Sciences, Isfahan, Iran

**Keywords:** cardiovascular diseases, exercise, global longitudinal strain, myocardial function, rehabilitation

## Abstract

Left ventricular global longitudinal strain (LVGLS) is a highly sensitive echocardiographic biomarker that detects signs of myocardial dysfunction. It has been proven that exercise-based cardiac rehabilitation (CR) improves LV-GLS but whether high-intensity interval training (HIIT) is more efficient than moderate-intensity interval training (MIIT) to improve LV-GLS as cardiac deformation index in cardiovascular patients is debatable. In the current systematic review and meta-analysis, different digital databases including PubMed, Scopus, Web of Science (ISI), and Google Scholar were searched systematically with no time restriction to answer the abovementioned question. Studies were included that reported GLS as the outcome in CVD subjects before and after enrolling in HIIT and/or MITT. A random effects model was used for meta-analysis. Eleven sets of results from nine articles—two of which had two sets of results—were included. The result of the sensitivity test to check the publication bias was not significant either for MIIT (*p*=0.211) or for HIIT (*p*=0.238). Our findings showed that GLS was improved significantly after both MIIT (−1.72. [−2.68, −0.77]) and HIIT (−1.86 [−3.01, −0.71]) in CVD patients; however, the effect of HIIT was greater than MIIT. Subgroup analysis results showed that baseline disease and duration of exercises do not influence the effect of training on GLS. More studies are needed to confirm the conclusion.

## 1. Introduction

Echocardiography is usually the first-line imaging modality that is readily available in the acute clinical setting. Left ventricular ejection fraction (LVEF) by echocardiography is a widely used parameter to describe myocardial performance after an acute myocardial infarction (AMI). The risk stratification after AMI is recommended 6–12 weeks after the admission, and LVEF < 35% is currently the major indication for an implantable cardiac defibrillator (ICD) implantation [1, 2].

Global longitudinal strain (GLS) is a promising echocardiographic indicator of cardiac performance in heart failure (HF) and can be easily assessed using a 2D echocardiogram [1, 3]. GLS primarily reflects the function of the subendocardial fibers, which are oriented longitudinally and are most susceptible to ischemic injury and wall stress. As a result, GLS can reveal abnormal contraction patterns even when LVEF remains within the normal range [1, 4, 5]. A review of data from 16 studies revealed that GLS is a more reliable predictor of major adverse cardiac events (MACE) compared to EF. The findings showed that changes in baseline GLS had a stronger association with mortality than LVEF [6].

Based on previous systematic reviews and meta-analyses of 63 studies, exercise-based CR reduces the rate of CV-related mortality while improving the quality of life and CV-modifiable risk factors [7, 8].

When it comes to enhancing cardiac function and performance, high-intensity interval training (HIIT) appears to be more effective than moderate-intensity training (MIIT) [9]. HIIT workouts typically start with a 5-minute steady-state warm-up of the muscles, followed by repetitions of HIIT (at 80%–90% of maximum heart rate) followed by recovery exercises of medium intensity [9]. MIIT included four sets of 4 min intervals at 55%–75% HR_max_ with a recovery period of 4 min at 45%–50% HR_max_, for a total of 42 min of exercise per session, including warm-up and cool-down [10]. Previous studies investigated the effect of exercise-based CR on LVEF [11–14]; however, the current systematic review sought to compare the effects of MIIT and HIIT on GLS in cardiovascular patients, with subanalyses conducted based on exercise duration and subject health conditions.

## 2. Methods

We conducted and reported this systematic review following the PRISMA 2021 statement [15]. The protocol for this review has not been registered with any organization. All the steps including searching, selection of final included papers, and quality assessment of articles were performed by two authors independently, and any discrepancies were resolved through discussion or consultation with a third reviewer.

### 2.1. Search Strategy

PubMed, Scopus, and Web of Science (ISI) were searched systematically of last 20 years using keywords: “cardiac rehabilitation,” physical activity, “exercise,” “rehabilitate⁣^∗^,” “global longitudinal strain,” “cardiac function,” “cardiovascular imaging,” and “left ventricular.” Only English studies were considered. The PICO was cardiovascular patients participated in CR/exercise-based CR/comparing outcomes before and after attending CR/GLS.

Below is our search strategy in PubMed as a sample:

((“cardiovascular patients” OR “coronary artery disease” OR “heart failure” OR “myocardial infarction”) AND (“moderate-intensity interval training” OR “MIIT” OR “high-intensity interval training” OR “HIIT” OR “exercise” OR “cardiac rehabilitation”) AND (“global longitudinal strain” OR “GLS” OR “left ventricular function” OR “myocardial strain”)) AND ((“Randomized Controlled Trial” [pt] OR “Clinical Trial” pt] OR “Comparative Study” [pt])).

### 2.2. Inclusion Criteria

Study design: randomized controlled trials (RCTs); population: patients diagnosed with cardiovascular diseases (e.g., coronary artery disease [CAD], HF, and myocardial infarction); intervention: studies involving either MIIT or HIIT; comparison: studies that compare MIIT with HIIT; outcome: measurement of GLS as a primary or secondary outcome. Studies published in English, published, peer-reviewed articles within the last 20 years (to ensure relevance and advances in exercise training and cardiovascular treatment) were included.

### 2.3. Exclusion Criteria

Nonrandomized studies, observational studies, case reports, reviews, editorials, and conference abstracts, studies involving healthy participants or populations without diagnosed cardiovascular disease, studies that do not specifically categorize training as either MIIT or HIIT or those involving other types of exercise interventions (e.g., resistance training and continuous moderate exercise), studies that do not report GLS as an outcome measure, studies published in languages other than English, unpublished manuscripts, dissertations, and gray literature were excluded.

### 2.4. Study Selection

Studies were included that reported GLS as an outcome in CVD subjects before and after enrolling in HIIT and/or MITT. Studies that only reported GLS status without mentioning the type of training (HIIT or MIIT) were not included. Subjects irrespective of sex or age had an AMI, revascularization (coronary artery bypass grafting [CABG] or percutaneous coronary intervention [PCI]), or who have angina pectoris or CHD defined by angiography, or those with CV risk factors such as hypertension. The EndNote reference management program was used to upload all references, which were then analyzed for deduplication, screening, and data extraction. Each article's full text was made available, and it was read in its entirety. The list of articles did not include reviews, case-control studies, conferences, or abstracts. No automation tools were utilized during the study selection process, aside from EndNote software, which was used solely for duplicate removal.

### 2.5. Data Extraction

Data regarding the authors, country and year of study, patient characteristics, GLS in MIIT and HIIT groups before and after CR, and duration of intervention were extracted using a standardized data collection form.

### 2.6. Statistical Methods

This study is conducted to determine the effect of MIIT and HIIT on the “GLS” by meta-analyses of studies. The final effect size was determined using the mean and standard deviation (SD) of “GLS” before and after MIIT and/or HIIT. As a summary result, mean changes with a 95% confidence interval (CI) were reported. *I*^2^ and *Q*-test are used to assess the heterogeneity between studies [16, 17]. Although the sensitivity analysis and the application of the Trim and Fill method indicated no publication bias in our study, a random effects model was used because this model often used in meta-analyses. This model assumes that the true effects vary between studies due to both within-study variance (sampling error) and between-study variance (heterogeneity). It provides a more conservative estimate by incorporating the additional variability. All analyses are performed using Stata 17 [18].

## 3. Results

### 3.1. Search

**Figure 1:**
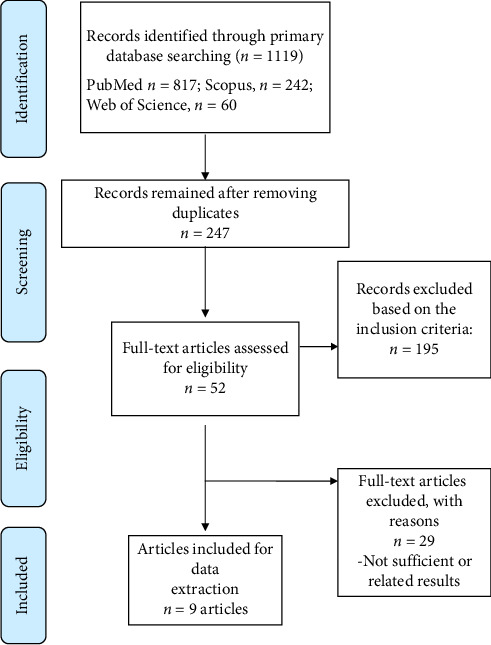
Flowchart of study selection.

Figure 1 illustrates the study selection flowchart, while Table 1 details the characteristics of each included study. Initially, 1199 articles were identified. After removing duplicates and excluding those that did not meet our inclusion criteria, nine articles comprising 11 sets of results were included in the analysis [19–27].

Two of the included articles focused on prehypertensive or hypertensive subjects [23, 24], three focused on post-AMI patients [21, 22, 27], one dealt with percutaneous coronary revascularization patients [26], one dealt with patients with preserved EF (HFpEF) [20], one dealt with patients with the acute coronary syndrome (ACS) [25], and one dealt with individuals with CAD [19]. Four studies reported data for both MIIT and HIIT [19–21, 25], three reported data for MIIT only [23, 24, 27], and three reported data for applying HIIT only [22, 26].

### 3.2. Meta-Analysis

#### 3.2.1. MII

**Figure 2:**
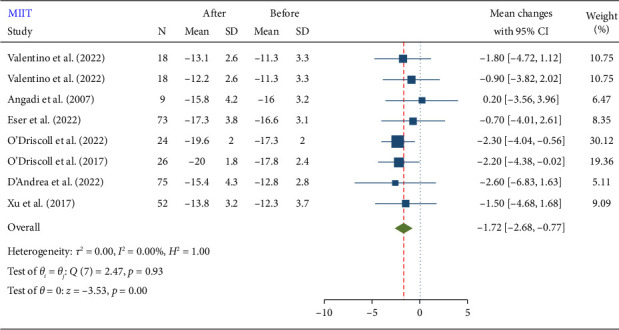
Forest plot of GLS changes after moderate-intensity interval training (MIIT).

According to the findings of this meta-analysis, MIIT resulted in a significant reduction in GLS 1.72 (−2.68, −0.77). The mean reduction in GLS after MIIT was −1.72, with a 95% CI ranging from −2.68 to −0.77 (Figure 2). This indicates that MIIT effectively improves GLS, which is a measure of cardiac function. The negative value reflects an improvement, as lower GLS values represent better myocardial deformation and thus better cardiac function. A summary of the results is presented in Table 2.

#### 3.2.2. HIIT

**Figure 3:**
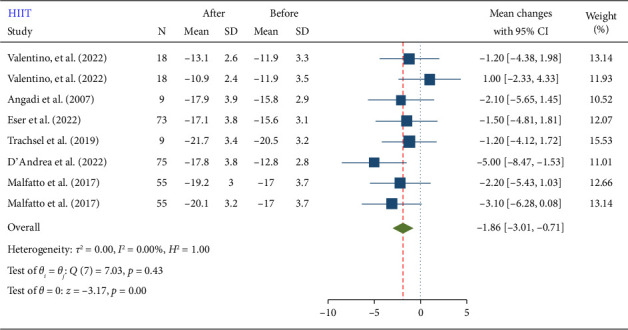
Forest plot of GLS changes after high-intensity interval training (HIIT).

HIIT also showed a significant reduction in GLS (−1.86 [−3.01, −0.71]) (Figure 3). The mean reduction in GLS after HIIT was −1.86, with a 95% CI ranging from −3.01 to −0.71. This suggests that HIIT is effective in enhancing cardiac function, similar to MIIT. The results showed that the reduction in GLS was more efficient with HIIT compared to MIIT. Specifically, the mean reduction in GLS was slightly larger for HIIT (−1.86) than for MIIT (−1.72). Although both forms of interval training significantly improve GLS, HIIT appears to have a more substantial impact. This difference, while relatively small, suggests that the higher intensity of HIIT might provide additional benefits for improving cardiac function in cardiovascular patients.

### 3.3. Subgroup Analysis

**Figure 4:**
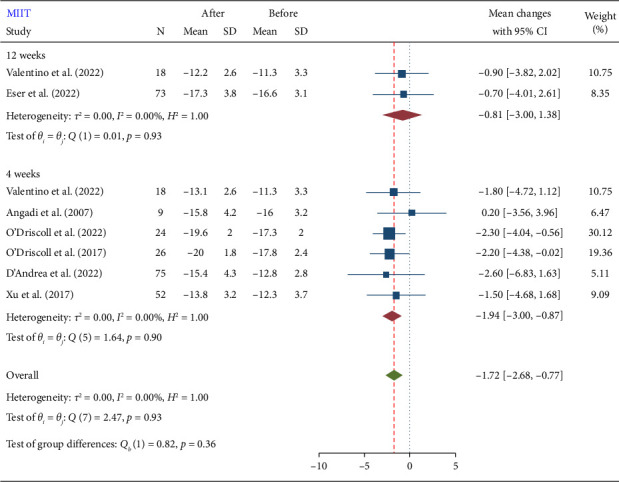
Forest plot of GLS subgroup analysis after moderate-intensity interval training (MIIT) in terms of training duration.

**Figure 5:**
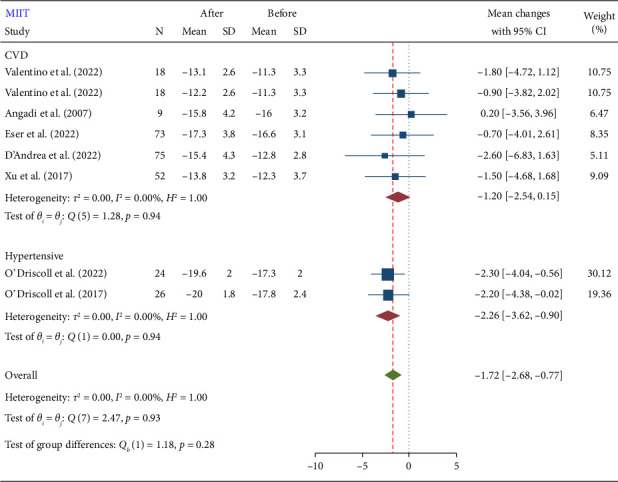
Forest plot of GLS subgroup analysis after moderate-intensity interval training (MIIT) in terms of the health condition of subjects.

**Figure 6:**
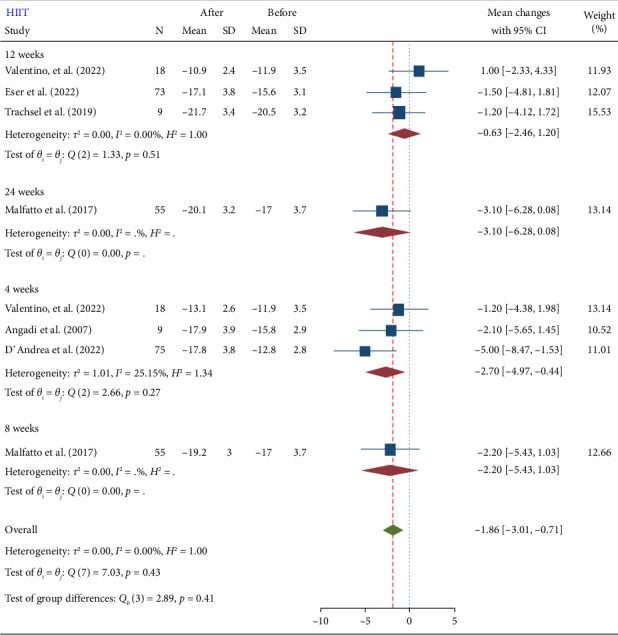
Forest plot of GLS subgroup analysis after high-intensity interval training (HIIT) in terms of training duration.

The test for subgroup differences (Figures 4, 5, and 6) was nonsignificant. Subgroup analysis results showed that baseline disease and duration of exercises do not influence the effect of training on GLS. However, due to the small number of included studies for subgroup analysis, the findings from subgroup analyses should be viewed as hypothesis-generating rather than conclusive. Therefore, while our subgroup analyses offer potential explanations for heterogeneity, these should be interpreted with caution and confirmed through larger, adequately powered studies. Our primary conclusions are based on the overall meta-analysis, accounting for heterogeneity using random effects models. A summary of the results is presented in Table 2.

### 3.4. Publication Bias Assessment

**Figure 7:**
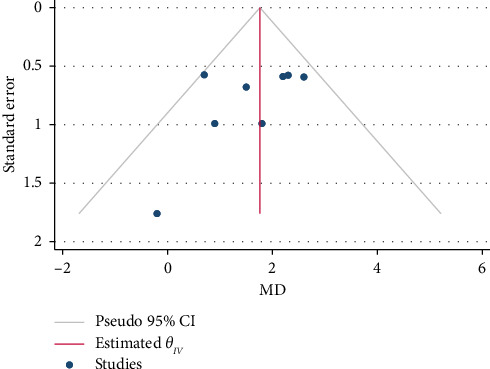
Funnel plot of publication bias in terms of GLS changes after moderate-intensity interval training (MIIT).

**Figure 8:**
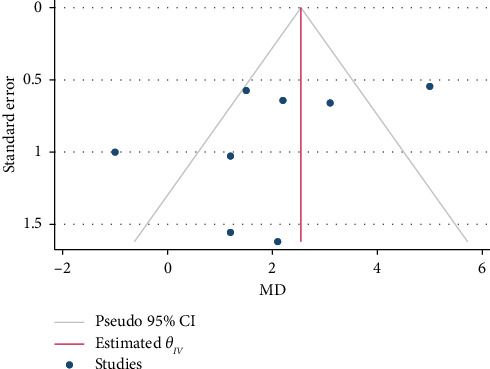
Funnel plot of publication bias in terms of GLS changes after high-intensity interval training (HIIT).

The result of the sensitivity test to check the publication bias was not significant either for MIIT (*p*=0.211) or for HIIT (*p*=0.238). The application of the Trim and Fill method did not alter the findings, thereby reinforcing the reliability of the meta-analysis results. The funnel plots can be seen in Figures 7 and 8.

### 3.5. Risk of Bias Assessment

**Figure 9:**
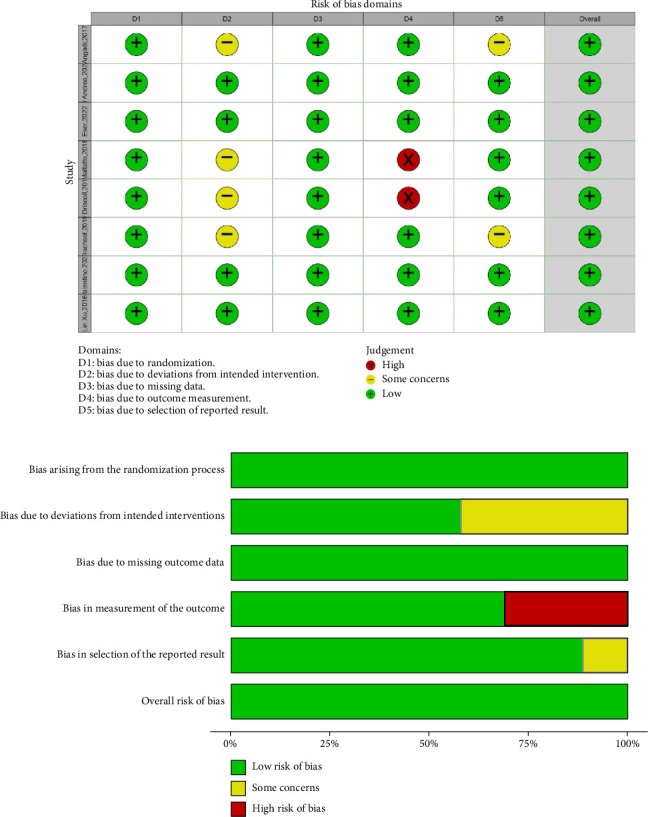
Risk of bias table to assess quality of the included studies: low risk of bias (green color), unclear risk of bias (blank), and high risk of bias (red color).

To assess the quality of the included RCTs, we used the risk of bias table. In this table (Figure 9), the bias of each study was evaluated in some area such as selection bias and performance bias etc. Out of eight studies, only two studies had reporting bias. In general, all studies were out of bias (high quality). This table was designed according to Review Manager 5.3.

## 4. Discussion

A very recently published systematic review and meta-analysis reported that exercise improved left ventricular GLS (LVGLS) significantly in CVD subjects [28]. In the present meta-analysis for the first time, we compared the effect of HIIT versus MIIT in improving cardiac deformation index as GLS in CVD patients. Our findings showed that GLS was improved significantly after both MIIT and HIIT in CVD patients; however, the effect of HIIT was more than MIIT. In terms of exercise duration and base-line health conditions, the subgroup analysis showed no significant differences.

The evaluation of LV function is crucial for many decisions, but EF has important restrictions when evaluating mild dysfunction, particularly when repeated evaluations are required. Speckle-tracking myocardial deformation imaging is widely available in contemporary echocardiography systems as a normal clinical tool that can measure GLS as a percentage [29]. Compared to LVEF, GLS appears to be a significant additive method for assessing LV function with better reproducibility [30]. A study by Karlsen et al. on 47 ACS patients showed that GLS is a more reproducible method for the evaluation of LV function than LVEF regardless of echocardiographic training [30].

The normal range of LVGLS has now been defined and reported as −18% and lower (i.e., more negative), abnormal as −16% or higher (i.e., less negative), with −16% to −18% being borderline [29]. The American Society of Echocardiography declared a score of −20 ± 2% for GLS to be normal [31].

Physical activity and aerobic capacity are linked to a lower risk of CVD and CVD-related mortality. Aerobic exercise is thus strongly advised for CVD patients to improve their cardiovascular health and reduce the risk of mortality [32, 33]. According to a bibliometric analysis study, the long-term HIIT interventions have positive effects on patient's ability to take care of themselves and their quality of life, and when HIIT and MIIT were compared, HIIT is more effective, safe, and well-tolerated by patients [34, 35]. Rognmo et al. previously found that exercise training reduces the risk of CVD-related and all-cause mortality in both male and female subjects with established CHD, particularly when exercising at moderate (MIIT) or high intensity (HIIT) in comparison with low exercise intensity [36].

The previous research has showed that LVEF and functional capacity are not related [37, 38]. In a study by Maia et al. in patients with systolic HF subjected to the cardiopulmonary rehabilitation program, the GLS has significantly associated with all functional cardiopulmonary exercise test (CPET) parameters including maxVO2 and T1/2VO2 [39]. In terms of functional capacity, it appears that GLS is more accurate than LVEF in classifying patients with HF [39]. Their research showed that the GLS could identify patients whose VO2 recovery was taking longer than expected. The lower the value of GLS, the greater the time required for the postphysical effort VO2 to be reduced to half [39].

Eser et al. in a study on STEMI patients who underwent MIIT and HIIT found that GLS and cardiorespiratory fitness improved significantly in both HIIT and MIIT groups at the end of CR and also at 1 year follow-up in HIIT and MIIT patients; however, further research is necessary because the GLS improvement in the HIIT group was significantly less than that in the MIIT group at the 1-year follow-up [21]. According to their findings, higher HIIT doses are unlikely to have improved LV remodeling; instead, they may have worse effects on GLS in patients who have recently undergone acute STEMI [21].

Angandi et al. studied the effect of HIIT (4 × 4 min, 85%–90% peak HR, interspersed with 3 min of active recovery) and MIIT (30 min at 70% peak HR) in patients with HFpEF. Patients were instructed 3 days per week for 4 weeks and had VO (2peak) testing and 2D echocardiography at the start and end of the 12 sessions of monitored exercise program. Right ventricular GLS (RVGLS) average improved significantly in the HIIT group after training, while RV-GSR, LV-GLS, and LV-GSR did not [20]. No significant improvements were observed following MIIT [20]. The study by Xu et al. on 52 AMI patients underwent MIIT for 4 weeks reported significant improvements in terms of GLS, GRS, GAS, GCS, and also LVEF [27]; however, Valentino et al. investigated the cardiovascular response to 12 week MIIT or stair climbing–based HIIT and discovered no significant improvement in any clinical measure of cardiovascular function, including GLS, with the exception of a small increase in cardiac apical rotation, which may indicate an early change in cardiac function [19].

Here are potential limitations for the study. First that despite efforts to identify all relevant RCTs, the number of studies included in the meta-analysis might be limited. A small sample size can affect the precision and generalizability of the findings. Second, despite attempts to minimize publication bias, there might be a tendency for studies with significant or positive results to be published, leading to an overestimation of the treatment effect. The meta-analysis might include studies that use different techniques to measure GLS, which could introduce variability in the results. The findings of the meta-analysis might not be generalizable to all cardiovascular patient populations, as the included studies might have focused on specific patient groups or settings.

## 5. Conclusion

It can be concluded that both HIIT and MIIT have significant effects in improving GLS; however, the effect of high-intensity training was greater. Subgroup analysis results showed that baseline disease and duration of exercises do not influence the effect of training on GLS. More studies are needed to confirm the conclusion.

## Figures and Tables

**Table 1:** Characteristics and extracted data of each study.

Authors/year/country	Population	Number/sex (male)	Training duration and program	Outcomes
Valentino et al., 2022, Canada [19]	CVD patients	18 (9 and 9) (8 M)	HIIT and MIIT 4 weeks	GLS was improved followed by HIIT and MITT but not significantly Cardiac apical rotation was improved slightly
Valentino et al., 2022, Canada [19]	CVD patients	18 (9 and 9) (8 M)	HIIT and MIIT 12 weeks	GLS was improved followed by HIIT and MITT but not significantly Cardiac apical rotation was improved significantly
Angadi et al., 2017, USA [20]	Patients with HFpEF	9 (8 M)	HIIT and MIIT 4 weeks	GLS was improved followed by HIIT but not by MITT
Eser et al., 2022, Switzerland [21]	Post-STEMI patients	73 (M)	HIIT and MIIT 12 weeks	GLS was improved followed by HIIT and MITT at the end of CR; however, there was a significantly smaller improvement in GLS at 1 year follow-up in the HIIT compared to the MIIT group
Trachsel et al., 2019, Canada [22]	Post-AMI patients	9	HIIT 4 weeks	GLS was improved followed by HIIT but not significantly
O'Driscoll et al., 2017, UK [23]	Prehypertensive	26 M	MIIT 4 weeks	GLS, SBP, DBP, and LV end systolic diameter significantly decreased after MIIT, but heart rate did not
O'Driscoll et al., 2022, UK [24]	Hypertensive	24 M	MIIT 4 weeks	MIIT improved GLS and global work efficiency and significantly reduced global wasted work
D'Andrea et al., 2022, Italy [25]	ACS	75 M	HIIT and MIIT 4 weeks	GLS, LA strain, and MW efficiency improved significantly after HIIT and were associated with functional capacity during the effort
Malfatto et al., 2017, Italy [26]	Percutaneous coronary revascularization patients	55	HIIT 8 weeks	HIIT increased peak VO (2) and improved GLS% and LVEF. The improvement persisted at 6 months
Malfatto et al., 2017, Italy [26]	Percutaneous coronary revascularization patients	55	HIIT 24 weeks	HIIT increased peak VO (2) and improved GLS% and LVEF. The improvement persisted at 6 months
Xu et al., 2017, China [27]	Post-AMI patients	52	MIIT 4 weeks	MIIT group showed significant improvements in terms of GLS, global radial strain (GRS), global area strain (GAS), global circumferential strain (GCS), and left ventricular ejection fraction (LVEF))

**Table 2:** Summary of the results.

	*N*	MD (95% CI)	*p* value^I^	*p* value ^II^
MIIT	8	−1.72 (−2.68, −0.77)	< 0.001	0.211
Duration				
4 weeks	6	−1.94 (−3, −0.87)	0.36	
12 weeks	2	−0.81 (−3, 1.38)		
Statues				
CVD	6	−1.20 (−2.54, 0.15)	0.28	
Hypertension	2	−2.26 (−3.62, −0.90)		
HIIT	8	−1.86 (−3.01, −0.71)	< 0.001	0.238
Duration				
4 weeks	3	−2.70 (−4.97, −0.44)	0.41	
8 weeks	1	−2.20 (−5.43, 1.03)		
12 weeks	3	−0.63 (−2.46, 1.20)		
24 weeks	1	−3.10 (−6.28, 0.08)		

^I^ For test (MD = 0).

^II^ For publication bias.

## Data Availability

All data generated during this study are included within this published article.

## Nomenclature

AMI: Acute myocardial infarction
BMI: Body mass index
CAG: Coronary angiography
CO: Cardiac output
CR: Cardiac rehabilitation
CV: Cardiovascular
CVD: Cardiovascular disease
DBP: Diastolic blood pressure
GAS: Global area strain
GCS: Global circumferential strain
GLS: Global longitudinal strain
GRS: Global radial strain
HF: Heart failure
HIIT: High-intensity interval training
HR: Heart rate
HRmax: Maximum heart rate
LV: Left ventricular
LVEF: Left ventricular ejection fraction
MACE: Major adverse cardiac events
MIIT: Moderate-intensity interval training
PCI: Percutaneous coronary intervention
SBP: Systolic blood pressure
